# Genome-Wide Association Mapping of Stem Rust Resistance in *Hordeum vulgare* subsp. *spontaneum*

**DOI:** 10.1534/g3.117.300222

**Published:** 2017-08-30

**Authors:** Ahmad H. Sallam, Priyanka Tyagi, Gina Brown-Guedira, Gary J. Muehlbauer, Alex Hulse, Brian J. Steffenson

**Affiliations:** * Department of Plant Pathology, University of Minnesota, St. Paul, Minnesota 55108; † Department of Crop and Soil Sciences, North Carolina State University, Raleigh, North Carolina 27695; ‡ United States Department of Agriculture-Agricultural Research Service, Raleigh, North Carolina 27695; § Department of Plant and Microbial Biology, University of Minnesota, St. Paul, Minnesota 55108; ** Department of Agronomy and Plant Genetics, University of Minnesota, St. Paul, Minnesota 55108

## Abstract

Stem rust was one of the most devastating diseases of barley in North America. Through the deployment of cultivars with the resistance gene *Rpg1*, losses to stem rust have been minimal over the past 70 yr. However, there exist both domestic (QCCJB) and foreign (TTKSK aka isolate Ug99) pathotypes with virulence for this important gene. To identify new sources of stem rust resistance for barley, we evaluated the Wild Barley Diversity Collection (WBDC) (314 ecogeographically diverse accessions of *Hordeum vulgare* subsp. *spontaneum*) for seedling resistance to four pathotypes (TTKSK, QCCJB, MCCFC, and HKHJC) of the wheat stem rust pathogen (*Puccinia graminis* f. sp. *tritici*, *Pgt*) and one isolate (92-MN-90) of the rye stem rust pathogen (*P. graminis* f. sp. *secalis*, *Pgs*). Based on a coefficient of infection, the frequency of resistance in the WBDC was low ranging from 0.6% with HKHJC to 19.4% with 92-MN-90. None of the accessions was resistant to all five cultures of *P. graminis*. A genome-wide association study (GWAS) was conducted to map stem rust resistance loci using 50,842 single-nucleotide polymorphic markers generated by genotype-by-sequencing and ordered using the new barley reference genome assembly. After proper accounting for genetic relatedness and structure among accessions, 45 quantitative trait loci were identified for resistance to *P. graminis* across all seven barley chromosomes. Three novel loci associated with resistance to TTKSK, QCCJB, MCCFC, and 92-MN-90 were identified on chromosomes 5H and 7H, and two novel loci associated with resistance to HKHJC were identified on chromosomes 1H and 3H. These novel alleles will enhance the diversity of resistance available for cultivated barley.

Among the many described fungal diseases of cultivated barley (*Hordeum vulgare* subsp. *vulgare* L.), stem rust ranks among the most devastating. The disease has been reported in many areas across the world, but is most important in the northern Great Plains of the USA and Canada as well as in northeastern Australia and eastern Africa (Dill-Macky *et al.* 1991; Mwando *et al.* 2012; Steffenson 1992). Yield losses of up to 58% have been reported for the crop in addition to marked reductions in malting quality traits (Dill-Macky *et al.* 1991; Mwando *et al.* 2012).

Barley can be infected by two stem rust pathogens: *Puccinia graminis* Pers.:Pers. f. sp. *tritici* Eriks and E. Henn. (*Pgt*) (the wheat stem rust fungus) and *P. graminis* Pers.:Pers. f. sp. *secalis* Eriks and E. Henn. (*Pgs*) (the rye stem rust fungus). The former is far more important than the latter across most major production areas. Breeding for stem rust resistance became a target trait in North American barley programs after the severe epidemics of the 1930s (Steffenson 1992). Since the mid-1940s, losses to stem rust in barley have been minimal, due largely to the resistance conferred by a single resistance gene, Reaction to *Puccinia graminis* 1 (*Rpg1*), in widely grown cultivars (Steffenson 1992). However, isolates of both *Pgt* and *Pgs* with virulence for *Rpg1* have been reported in North America (Martens *et al.* 1989; Roelfs *et al.* 1993; Steffenson 1992). In one case, *Pgt* pathotype QCC (now designated QCCJB) caused scattered losses on barley cultivars with *Rpg1* in both the Upper Midwest region of the USA and eastern Prairie Provinces of Canada (Harder and Dunsmore 1993; Roelfs *et al.* 1993) from 1989 to 1992, but is rarely found now. A new extant threat to barley production in North America is *Pgt* pathotype TTKSK (isolate synonym Ug99), which was first found in Uganda in 1998 and race typed in 1999 (Pretorius *et al.* 2000). This pathotype is particularly noteworthy because it possesses virulence for *Sr31*, a widely used and effective stem rust resistance gene in wheat. Pathotype TTKSK is a major threat to wheat production because 90–95% of the global crop hectarage is susceptible (Singh *et al.* 2006, 2011). Similarly, in an extensive evaluation of over 1900 cultivated barley accessions from around the world, over 95% were found susceptible (Steffenson *et al.* 2017). The large percentage of cultivated wheat and barley germplasm susceptible to a single pathotype of *Pgt* is both exceptional and extremely dangerous with respect to food security.

In barley, eight stem rust resistance genes with Mendelian inheritance have been described. The first one discovered was *Rpg1* from the Swiss landraces Chevron (CIho 1111) and Peatland (CIho 5267). Although this gene provided durable resistance for a long time and is still widely effective against many pathotypes of *Pgt* (Steffenson 1992), it is ineffective against QCCJB and TTKSK as well as some isolates of *Pgs* (Jin *et al.* 1994; Kleinhofs *et al.* 2009; Steffenson *et al.* 2016). *Rpg2* and *Rpg3* were derived from Heitpas-5 (CIho 7124) and GAW 79-3 (PI 382313), respectively, and confer adult plant resistance (Patterson *et al.* 1957; Jedel *et al.* 1989; Jedel 1990; Sun and Steffenson 2005; Case 2017). The *rpg4*/*Rpg5* complex was first described from line Q21861 (PI 584766) and consists of two closely linked but distinct loci: the *rpg4*-mediated resistance loci (RMRL) RMRL1 and RMRL2. The former locus contains three distinct genes, *Rpg5* and *HvRga1*, encoding leucine-rich repeat (LRR) proteins, and *HvAdf3*, an actin depolymerizing factor-like gene, and the latter locus at least one gene involved in conferring resistance (Wang *et al.* 2013). The *rpg4*-mediated resistance acts in a recessive manner against a number of important pathotypes including QCCJB and TTKSK. In fact, it is the only gene/gene complex described to date in barley that provides highly effective all-stage resistance against the dangerous African stem rust pathotype of TTKSK and its variants. Further research established that *Rpg5* alone confers dominant resistance to some isolates of *Pgs* (Sun *et al.* 1996). The *rpg4*-mediated resistance is very temperature sensitive. At 18–20°, barleys carrying the gene complex are highly resistant to pathotypes such as QCCJB and TTKSK, whereas at temperatures exceeding 27° these same barleys are rendered susceptible (Jin *et al.* 1994; Sun *et al.* 1996). Other stem rust resistance genes described from barley include *RpgU* discovered subsequent to *Rpg1* in Peatland (Fox and Harder 1995); *rpg6*, a recessive-acting gene in 212Y1, a barley line that carries an introgression of *H. bulbosum* L. chromatin (Fetch *et al.* 2009); and *rpgBH*, a recessive gene from Black Hulless (CIho 666) that confers resistance to *Pgs* (Steffenson *et al.* 1984; Sun and Steffenson 2005).

Given the reports of virulence for the major stem rust resistance genes carried in widely grown barley cultivars, it is imperative that additional resistance sources be identified. Several investigations of cultivated barley germplasm revealed that stem rust resistance to pathotypes QCCJB and TTKSK is very rare (Jin *et al.* 1994; Zhou *et al.* 2014; Steffenson *et al.* 2013, 2017; Case 2017). One readily accessible source of genetic diversity for barley breeding is the wild progenitor *H. vulgare* subsp. *spontaneum*. This subspecies originated in the Fertile Crescent area, can hybridize readily with cultivated barley, and is widely known as a rich source of genetic diversity for many traits, especially disease and pest resistance (Fetch *et al.* 2003; Ames *et al.* 2015; Armstrong *et al.* 2016). To capture a portion of the diversity present in *H*. *vulgare* subsp. *spontaneum* in a practical number of accessions, the Wild Barley Diversity Collection (WBDC) was developed. This collection consists of 318 accessions selected on the basis of various ecogeographic characters. Most of the WBDC accessions are from the Fertile Crescent region where the diversity of the subspecies is the highest and large populations are common; however, representative samples were also included from Central Asia, South Central Asia, North Africa, and the Caucasus region (Steffenson *et al.* 2007).

Genome-wide association studies (GWAS) are an efficient means for mapping multiple traits in a single population, *e.g.*, a wild germplasm panel. This method is ideally suited for identifying favorable genes in natural populations and is being widely used today in many crop plants for such diverse traits as yield, quality, disease resistance, and abiotic stress tolerance (Skøt *et al.* 2005; Quesada *et al.* 2010; Rosenberg *et al.* 2010). Compared to mapping in biparental populations, GWAS has a number of advantages including: (i) the utilization of existing populations, thereby preempting the need to develop multiple biparental populations that require several years of development and a large labor effort; (ii) a greater capacity for detecting more alleles since the panel consists of diverse accessions rather than a single segregating population whose parents differ for only one or a few target traits; and (iii) a higher resolution power for quantitative trait locus/loci (QTL) detection due to numerous recombinations in the population, particularly in panels of wild species with a long evolutionary history of such events. One critical aspect that must be resolved with GWAS is the issue of population structure, which is the mixture of several subpopulations within an association mapping panel. This problem can be overcome by incorporating population structure, genetic relatedness, or both of these components into the mixed model (Yu *et al.* 2006; Kang *et al.* 2008; Bradbury *et al.* 2011).

Our long-term goal is to catalog and characterize economically important genes in *H*. *vulgare* subsp. *spontaneum* for cultivated barley improvement. The objectives of this research were to: (1) evaluate a diverse collection of wild barley accessions for resistance to several *P. graminis* cultures, including those virulent for *Rpg1* at the seedling stage; and (2) utilize GWAS to precisely position identified resistance loci based on the recently completed reference genome sequence of barley (Mascher *et al.* 2017).

## Materials and Methods

### Plant materials

The WBDC consists of 318 accessions of *H*. *vulgare* subsp. *spontaneum* selected on the basis of various ecogeographic characters such as longitude/latitude, elevation, temperature, rainfall, soil type, *etc*. Of the 318 WBDC accessions, four were not included in the study due to poor seed germination. Wild barley originated in the Fertile Crescent where many large populations still exist today. Thus, most of the accessions (*N* = 250; 78.6%) selected for the WBDC are from this region. However, the habitat range of barley’s wild progenitor is more extensive so representative samples were also included from North Africa (*N* = 8; 2.5%), the Caucasus region (*N* = 9; 2.8%), Central Asia (*N* = 41; 12.9%), and South Central Asia (*N* = 9; 2.8%). Single plant selections were made from the raw *H*. *vulgare* subsp. *spontaneum* accessions and selfed at least twice before being phenotyped for stem rust reaction and at least three times before being genotyped. In addition to the WBDC, the resistant controls of Chevron, carrying the stem rust resistance gene *Rpg1*; Q21861, carrying genes *Rpg1* and *rpg4/Rpg5*; and Q/SM20, a doubled haploid line derived from Q21861 that carries only *rpg4*/*Rpg5* (Steffenson *et al.* 1995) were included. *Rpg1* and *rpg4/Rpg5* are the most common stem rust resistance genes being used in commercial barley breeding programs and are readily detected by seedling phenotyping to selected *Pgt* pathotypes and also by genotyping (Steffenson *et al.* 2017). Hiproly (PI 60693), a barley accession that lacks stem rust resistance, was used as the susceptible control in all stem rust experiments.

### Pathogen cultures and molecular assays for the Rpg5 and Rpg1 stem rust resistance genes

Four pathotypes of *Pgt* and one isolate of *Pgs* were used to assess the resistance spectrum of the WBDC accessions. QCCJB (isolate QCC-2) and TTKSK (04KEN156/04) represent a domestic (North Dakota) and foreign (Kenya) *Pgt* pathotype, respectively, with virulence for the resistance gene *Rpg1*, and 92-MN-90 is a domestic (Minnesota) *Pgs* isolate with virulence for this gene. MCCFC (isolate A-5) is a *Pgt* pathotype that can differentiate barley accessions carrying *Rpg1* from those that lack any resistance gene, whereas pathotype HKHJC (isolate CRL-1) is a *Pgt* pathotype that can selectively detect accessions carrying *Rpg1* in the presence of other *Rpg* genes (Mirlohi *et al.* 2008; Sun and Steffenson 2005). HKHJC is unique in that it is one of the few known *Pgt* pathotypes with virulence for the *rpg4*/*Rpg5* complex (Steffenson *et al.* 2016). These five *P*. *graminis* cultures represent diverse virulence types for assessing the resistance spectrum of the WBDC and may uncover new genes for enhancing the resistance of cultivated barley. The *rpg4/Rpg5* gene complex is very effective against pathotype TTKSK (and also QCCJB, MCCFC, and 92-MN-90); thus, accessions exhibiting resistance to this pathotype may carry this gene complex. To corroborate this possibility, TTKSK-resistant accessions were subjected to a molecular assay for a functional *Rpg5* gene, described in detail by Steffenson *et al.* (2017). Resistance to pathotype HKHJC may indicate the presence of *Rpg1* in barley. Thus, any WBDC accession exhibiting a resistant reaction to this pathotype was subjected to a molecular assay for *Rpg1*. This assay was conducted according to the methods of Eckstein *et al.* (2003) as modified by Derevnina *et al.* (2014).

### Plant growth conditions, rust inoculations, and incubation conditions

Seeds of the WBDC accessions and controls were planted in plastic pots (7.6 × 7.6 × 10.8 cm l × w × h) filled with a 50:50 mixture of steam-sterilized native soil: Metro-Mix 200 growing media (Sun Grow Horticulture, Quincy, MI) and fertilized with Osmocote 14-14-14 (Scott’s Co., Marysville, OH; 1.4 g per pot) and Peters Dark Weather 15-0-15 (Scott’s Co., 150 g per gallon at 1/16 dilution) formulations. To break possible residual dormancy and encourage uniform germination and emergence, seeds were incubated at 4° for 1 wk and then transferred to a growth chamber at 20–22° with 14 hr photoperiod (provided by 160-W fluorescent and 60-W incandescent lamps emitting 150–250 μmol photons s^−1^ m^−2^). For the evaluations to pathotype TTKSK, plants were grown, inoculated, and incubated inside the Minnesota Department of Agriculture/Minnesota Agricultural Experiment Station Biosafety Level-3 (BSL-3) Containment Facility on the University of Minnesota St. Paul campus under a temperature regime of 19–22° and 14- to 16-hr photoperiod (400-W high-pressure sodium lamps emitting a minimum of 300 μmol photons s^−1^ m^−2^) (Steffenson *et al.* 2017). Postinoculation conditions for the WBDC varied depending on the rust culture used and the resistance genes targeted for detection. For pathotype QCCJB, accessions were incubated at 18–21°, a regime for which the gene complex of *rpg4/Rpg5* is especially effective and for HKHJC at 22–25°, a range under which the gene *Rpg1* is highly effective. Postinoculation conditions for plants infected with pathotype MCCFC and isolate 92-MN-90 were the same as those described for HKHJC. Detailed methods on the increase, storage, and preparation of, and inoculation with rust cultures were as previously described (Steffenson *et al.* 2017). Fully expanded first leaves of seedling plants were inoculated with the urediniospore suspensions using a custom rust inoculator pressured at 25–30 kPa (Sun and Steffenson 2005). A concentration of 5 mg urediniospores/0.7 ml of mineral oil (Soltrol 170; Phillips Petroleum, Bartlesville, OK) was used with rust cultures QCCJB, MCCFC, and 92-MN-90. For TTKSK, a concentration of 15 mg urediniospores/0.7 ml of mineral oil was used, reflecting the different infectivity rate inside the BSL-3 facility (Steffenson *et al.* 2017). The oil carrier was allowed to evaporate from the leaf surfaces for several hours; then, plants were placed in mist chambers under the conditions previously described (Steffenson *et al.* 2017; Sun and Steffenson 2005).

### Disease assessment

Stem rust Infection Types (ITs) were assessed 12–14 d after inoculation based on the 0–4 rating scale originally developed for wheat by Stakman *et al.* (1962) and modified for barley by Miller and Lambert (1955). For the purpose of GWAS, the categorical phenotype data were converted into numeric data as described previously (Zhou *et al.* 2014). Briefly, IT “0” was assigned a value of 0.0, IT “0;” a value of 0.5, IT “1” a value of 2.0, IT “2” a value of 3.0, IT “3−” a value of 3.5, IT “3” a value of 4.0, IT “3+” a value of 4.5, and IT “4” a value of 5. These numeric phenotype values capture more quantitative variation in the subtle categories of the observed ITs and reflect biologically and epidemiologically relevant differences in the diverse host–parasite interactions (Zhou *et al.* 2014). The final phenotype was represented by a coefficient of infection (CI), which was calculated by multiplying the numeric phenotypic value times a general proportion value of the respective ITs observed on each plant according to the formulae described by Zhou *et al.* (2014). Disease assessments were made on the WBDC in two to three separate experiments conducted in a completely randomized design with repeated controls. Accessions giving variable reactions across replicates were repeated an additional time. The mean CI calculated over all experiments was used in GWAS. Barley accessions are often classified into general reaction groups based on the following categories of observed raw ITs: 0 or 0; as highly resistant, 1 as resistant, 2 as moderately resistant, 3− as moderately susceptible, and 3, 3+, or 4 as susceptible (Steffenson *et al.* 2017). In this study, the WBDC was classified into just two categories: resistant, where the mean CI was ≤2.7 (equivalent to a raw IT of 2 or lower) and susceptible, where the mean CI was >2.7 (equivalent to a raw IT of 23− or higher).

### Genotypic evaluation

The WBDC accessions were genotyped using Genotyping by Sequencing (GBS) technology. DNA was extracted using the Mag-Bind Plant DNA Plus kit from Omega Bio-tek (Norcross, GA), following the manufacturer’s instructions. Genomic DNA was quantified using the Quant-iT PicoGreen dsDNA Assay Kit and normalized to 20 ng µl^−1^. GBS libraries were created using the *Pst1-Msp1* restriction enzyme combination as described in Poland *et al.* (2012). The samples were pooled together at 48-plex to create pooled libraries, which were then sequenced on Illumina Hi-Sequation 2500.

Single nucleotide polymorphism (SNP) calling was performed with the TASSEL 5 GBSv2 pipeline (https://bitbucket.org/tasseladmin/tassel-5-source/wiki/Tassel5GBSv2Pipeline) using a 64-base kmer length and minimum kmer count of five. Reads were aligned to the reference sequence “150831_barley_pseudomolecules.fasta” (http://webblast.ipk-gatersleben.de/barley_ibsc/downloads/; Mascher *et al.* 2017) using the aln method of the Burrows–Wheeler aligner, version 0.7.10 (Li and Durbin 2009). Raw SNP data generated from the TASSEL pipeline in vcf format were then filtered to remove taxa with >90% missing data and sites with genotype quality <30, ultimately yielding 213,825 markers of which 208,490 have chromosomal positions. Genotypic data were then filtered to select biallelic SNPs with minor allele frequency (MAF) ≥0.05, missing data ≤20%, and heterozygosity ≤5%. These filtering steps resulted in a total of 54,118 SNP markers. Marker names were assigned based on their chromosome location and base pair position (bp) on the barley reference genome (Mascher *et al.* 2017). Genotypic data for the WBDC will be deposited in The Triticeae Toolbox (T3) (https://triticeaetoolbox.org/).

### Characterization of linkage disequilibrium, population structure, genetic distance, and kinship

Adjacent pairs of markers, as well as a sliding window of 25 adjacent markers, were used to characterize linkage disequilibrium (LD) in the WBDC as *r^2^* using TASSEL v5 (Bradbury *et al.* 2007). From 54,118 total markers, 3276 were removed because they were in perfect LD (*r^2^* = 1) with another marker. For genetic characterization and association mapping, 50,842 markers with defined map positions were identified. Adjacent marker LD was used to visualize the distribution of LD across chromosomes. LD estimates, expressed as *r^2^* and based on a sliding window of 50 markers throughout the genome, were calculated in TASSEL and plotted against the physical distance. Locally weighted scatter plot smoother (LOWESS) was run in JMP (JMP, Version 11.2, SAS Institute Inc., Cary, NC) to visualize the change of LD with physical distance.

To determine covariates for GWAS, Principal Component Analysis (PCA) was performed in R to characterize subpopulations within the WBDC using all 50,842 markers generated by GBS (R Development Core Team 2013). In addition to PCA, a Bayesian grouping method was used to determine the number of subpopulations. STRUCTURE software v 2.3.4 (Pritchard *et al.* 2000; Falush *et al.* 2007) was used to assess the optimal number of subpopulations (Q) using 4000 SNP markers that were randomly distributed across all barley chromosomes and in linkage equilibrium with each other. The number of subpopulations specified for this analysis ranged from Q = 2–10 and was modeled with a burn-in of 10,000 cycles followed by 10,000 iterations in three separate runs. A ΔQ value was calculated for the specified subpopulation numbers (Evanno *et al.* 2005) to obtain the most stable number for use in the final structure analysis. The most stable subpopulation number was used to perform additional runs within STRUCTURE, in this case with a burn-in of 20,000 cycles followed by 100,000 iterations in a single run. The admixture model was assumed in STRUCTURE with the use of correlated allele frequency. Genetic kinship was estimated as the realized additive relationship matrix in the rrBLUP package of R using all 50,842 markers (Endelman 2011; R Development Core Team 2013). The realized relationship matrix was estimated as:

$$K=\frac{WW^{\prime }}{2\sum p_{i}\left(\mathrm{1-}p_{i}\right)},$$

where *W = M – P*, *M* being the individuals by SNP loci marker matrix and *P* the frequencies of alleles expressed as *2*(*p_i_ – 0.5*) with *p_i_* representing the allele frequency of marker *i* (VanRaden 2008). The kinship relationship for all WBDC accessions was visualized as a heatmap in R (R Development Core Team 2013).

### Data analysis

Distributions of the phenotypic data for the WBDC to all *P*. *graminis* cultures were visualized using histograms. To achieve normally distributed data, the mean of the CI values were subjected to the log_10_, square, and square root transformations for the purpose of GWAS. The Shapiro–Wilk test was performed in JMP to check for normality of the transformed and untransformed CI means. Pearson correlation coefficients for pairwise comparisons between stem rust cultures were calculated using the mean CI in R (R Development Core Team 2013). Additionally, ANOVA was performed in R for all *P. graminis* cultures using the mean CI (R Development Core Team 2013). Variance components were estimated from the expected mean squares of the ANOVA. Broad-sense heritability (*H^2^*) for resistance to all *P*. *graminis* cultures in the WBDC was estimated on an entry mean basis using the equation:

$$H^{2}=\frac{\sigma_{g}^{2}}{\sigma_{g}^{2}+\left(\frac{\sigma_{e}^{2}}{n}\right)},$$

where *σ_g_^2^* is the genetic variance, *σ_e_^2^* is the error variance, and *n* is the number of replications. Comparison of subpopulation phenotype means was performed for each *P*. *graminis* culture using ANOVA. Mean separation tests were based on Tukey’s Honestly Significant Difference (HSD) with *α* = 0.05.

### GWAS

To identify markers associated with resistance to the five *P*. *graminis* cultures, GWAS was conducted for the WBDC accessions using three different models: (1) a mixed linear model that accounts for population structure (*Q*) + kinship (*K*), (2) a mixed linear model that accounts for *K* only, and (3) a linear model that accounts for background genetic effects (*G* model) (Yu *et al.* 2006; Kang *et al.* 2008; Bernardo 2013). In this investigation, the results from the mixed linear models were given priority; however, the *G* model was used to confirm the identified QTL. For the *Q + K* model, the mixed model is described as Y = mean + Wm + Qv + Zu + e where **Y** is the vector of the mean CI for each accession across replications, **m** is the vector of fixed SNP effect, **v** is the vector of fixed population effect, **u** is the vector of random genetic background effect for each accession, and **e** is the vector of residuals. W, Q, and Z are incidence matrices. The parameter **u** is distributed as N(0, K*σ^2^_g_*), where K is the kinship matrix and *σ^2^_g_* is the genetic variance. The parameter **e** is distributed as N(0, I*σ^2^_e_*) where I is the identity matrix and *σ^2^_e_* is the error variance. For the *K* model, the mixed model is described as Y = mean + Wm + Zu + e as described above. The first two principal components from the PCA analysis were used to account for population structure in the mixed model. The kinship relationship matrix, calculated as the realized additive relationship matrix based on all 50,842 markers, was used to account for genetic relatedness among WBDC accessions in the mixed model. In addition to these two models, we used the *G* model that accounts for background marker effects across the remaining chromosomes not being evaluated for marker–trait associations. The *G* model was implemented in three steps. First, genome-wide marker effects were calculated using RR-BLUP (Meuwissen *et al.* 2001). The estimated marker effects in the first step were used to adjust for CI means in chromosomes not being evaluated for marker–trait associations. Then, multiple regression was performed for each chromosome separately. The *G* model was found to be an effective model for detecting QTL (Schaefer and Bernardo 2013). To decrease the computational time for *G* model analysis and reduce multicollinearity between adjacent markers, LD for adjacent markers was used to remove one member (the one with more missing data) of the identified marker pairs with high LD (*r^2^* > 0.80). This step eliminated 15,959 markers, leaving 34,883 markers in total. *Q* + *K* and *K* association mapping models were implemented in the rrBLUP package in R (Endelman 2011; R Development Core Team 2013). The *G* model was implemented in a Fortran program (Bernardo 2013). Multiple testing comparisons for the *Q* + *K* and *K* models were accounted for using the False Discovery Rate, implemented in the q-value package in R at significance level of 0.05 to control for type I errors (Benjamini and Hochberg 1995; Storey and Tibshirani 2003; R Development Core Team 2013). To correct for *G* model multiple testing comparisons, the Bonferroni correction was used at an experimental-wise error of 0.01 with all of the 34,883 markers. The proportion of phenotypic variance explained by each marker (R^2^) was calculated as R^2^ = SS_reg_/SS_tot_, where SS_reg_ is the regression sum of squares and SS_tot_ is the total sum of squares of the regression model.

### Gene annotation

For each SNP found significant in the GWAS analysis, a 2-Mbp region around the SNP was investigated for the presence of high confidence and low confidence genes. Both gene annotations and POPSEQ positions were extracted from the reference assembly (Mascher *et al.* 2013, 2017). The gene annotation information for the barley reference genome is available at the IPK Barley BLAST Server (http://webblast.ipk-gatersleben.de/barley_ibsc/downloads/). Additionally, sequences for the resistance genes *Rpg1*, *rpg4*, and *Rpg5* were obtained from the National Center for Biotechnology Information (NCBI). These sequences were blasted against the barley genome assembly (Mascher *et al.* 2017) in the IPK server to determine the physical positions of these stem rust resistance genes. Alignment between known sequences was performed in the NCBI BLAST sequence alignment (Zhang *et al.* 2000).

### Data availability

Raw ITs for the five *P. graminis* cultures on the 314 WBDC accessions are provided in Supplemental Material, Table S1. Genotypic data will be available in T3 (https://triticeaetoolbox.org/).

## Results

### Genotypic analyses and linkage disequilibrium

**Figure 1:**
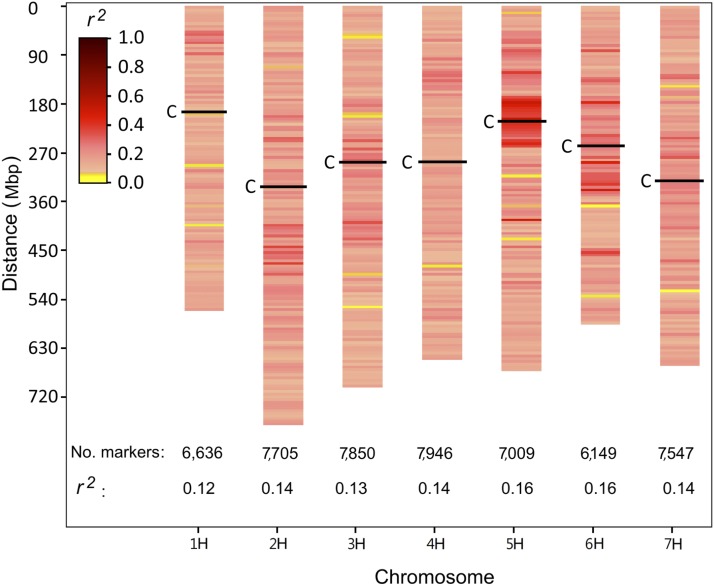
Heatmap for the distribution of linkage disequilibrium (LD) across the genome of *H. vulgare* subsp. *spontaneum* accessions of the WBDC estimated as *r^2^* using SNP markers, number of SNP markers for each chromosome, and average adjacent marker LD for each chromosome. The approximate positions of centromeres (C) are given for each chromosome.

The density and LD levels among markers are key factors for revealing the genetic architecture of a trait after proper accounting of genetic relatedness or population structure. Using the comprehensive and cost-effective genotyping method of GBS, 208,490 SNP markers with defined chromosomal and physical map positions were identified in the WBDC. The number and coverage of identified SNP markers enabled greater capture of genetic variability in the rust phenotypes for identifying significant marker–trait associations as well as enhanced analyses of LD, population structure, and genetic relatedness. After screening for a MAF of 5% and removing markers having perfect LD with each other, a total of 50,842 markers were used in the final analyses (Figure S1 in File S1). The percentage of missing genotypic data was 8.6% in this marker list. The distribution and estimates of LD for all chromosomes are displayed in Figure 1. The average LD (expressed as *r^2^*) for adjacent pairs of markers in individual chromosomes ranged from 0.12 (chromosome 1H) to 0.16 (chromosomes 5H & 6H) with an overall average marker LD of 0.14. Using a sliding window of 25 markers, the overall average marker LD across the genome was 0.10. LD in the WBDC was reduced to <0.10 in ∼0.2 Mbp using a sliding window of 50 markers across the genome (Figure S2 in File S1).

### Population structure and genetic relatedness in the WBDC

**Figure 2:**
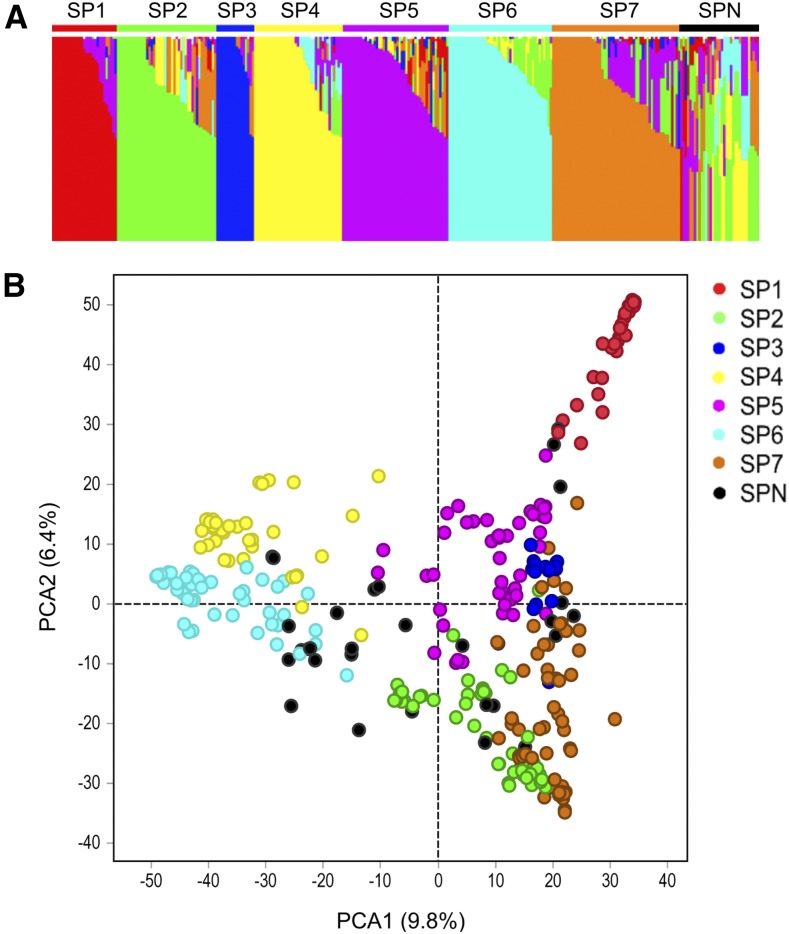
(A) Population structure of the WBDC inferred from Bayesian grouping implemented in STRUCTURE where the number of subpopulations (SP) identified was seven. (B) PCA of the WBDC identified seven major groups that corresponded closely with the subpopulation assignment results found with STRUCTURE analysis. Accessions not belonging to any of the seven subpopulations are shown in black.

**Figure 3:**
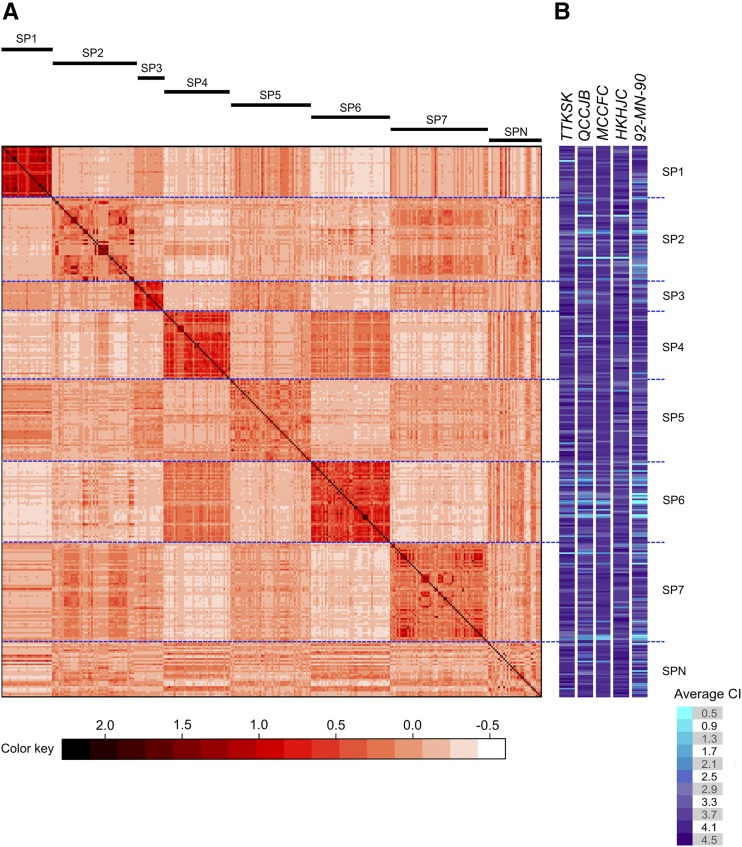
(A) Heatmap matrix displaying the genetic kinship among accessions of the WBDC calculated based on 50,842 SNP markers with corresponding subpopulations identified by STRUCTURE given above. (B) Heatmap for the average coefficient of infection (CI) of WBDC accessions to *P. graminis* f. sp. *tritici* pathotypes TTKSK, QCCJB, MCCFC, and HKHJC and *P. graminis* f. sp. *secalis* culture 92-MN-90 where lighter colors indicate higher resistance levels.

Using a random set of 4000 markers in linkage equilibrium across the barley genome, the WBDC accessions were classified into subpopulations using STRUCTURE analysis. Seven subpopulations were identified that varied in their allele frequencies (Figure 2A). In most cases, individual WBDC accessions were assigned to the same respective subpopulations based on both STRUCTURE and PCA analyses (Figure 2B). The amount of genotypic variation explained by PC1 and PC2 was 9.8 and 6.4%, respectively (Figure 2B). In addition to STRUCTURE and PCA, genetic kinship was calculated among WBDC accessions using all 50,842 markers, and the results for genetic relatedness (Figure 3A) were similar to those found in the other two analyses (Figure 2, A and B) in that seven subpopulations were identified. These seven subpopulations are distinguished by the darker red color in the heatmap matrix (Figure 3A). The kinship analysis revealed an especially close genetic relationship among accessions within subpopulations 1, 3, 4, 6, and 7 (Figure 3A).

**Figure 4:**
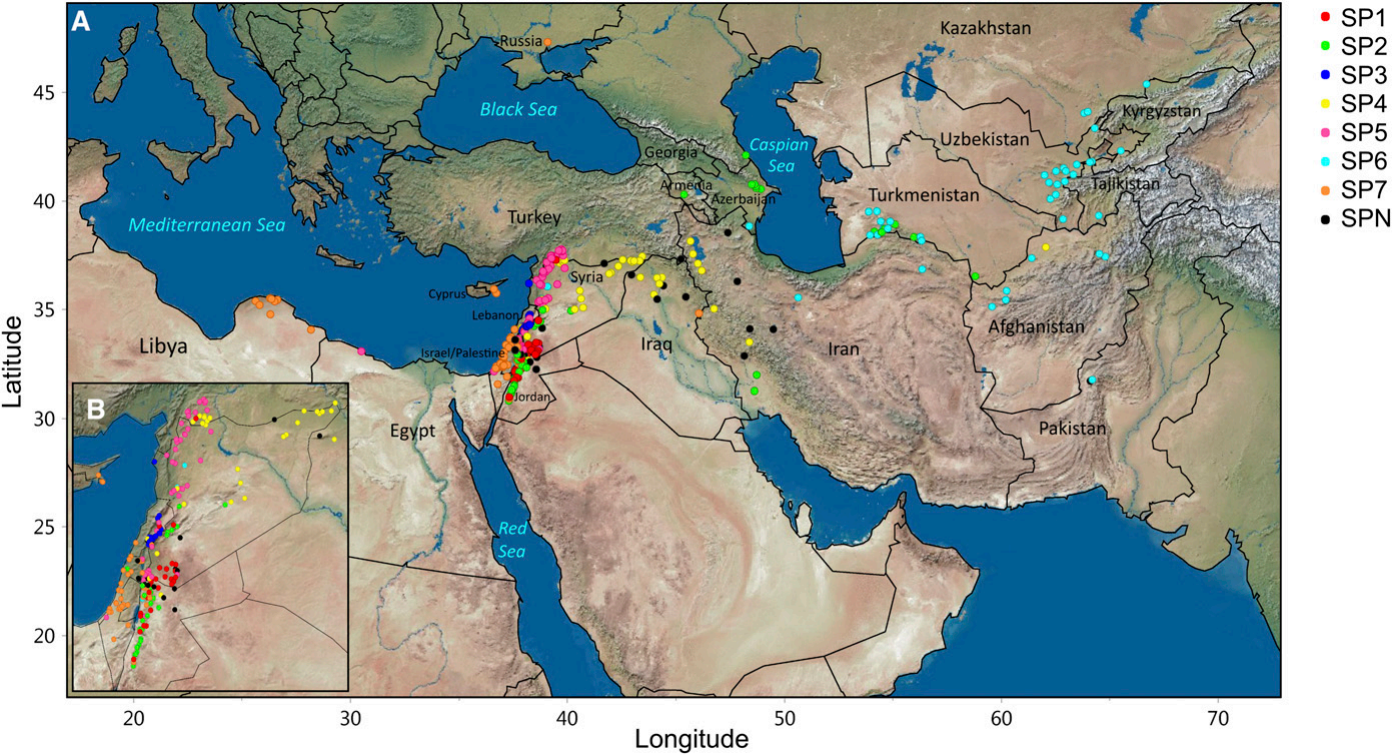
Geographic distribution of accessions in the WBDC and their subpopulation assignment as identified by structure analysis for (A) the entire region and (B) an enlarged insert of the Levant region.

Distinct geographic distributions were found for the subpopulations identified by STRUCTURE, PCA, and genetic kinship (Figure 4). Subpopulation 1 includes accessions mainly from southern Syria and western Jordan; subpopulation 2 includes accessions mainly from western Jordan, southwestern Syria, southwestern Iran, the Caucasus region (*i.e.*, Azerbaijan, Armenia, and Dagestan within Russia), and Turkmenistan; subpopulation 3 includes accessions chiefly from western Syria and eastern Lebanon; subpopulation 4 includes accessions chiefly from west Asia including northern and central regions of Syria, southeastern Turkey, northern Iraq, and northwestern Iran; subpopulation 5 includes accessions mainly from extreme northwestern Syria, southeastern Turkey, Jordan, Lebanon, northern Israel/Palestinian Authority Territories, and Egypt; subpopulation 6 includes accessions mostly from Central Asia (southwestern Turkmenistan, eastern Uzbekistan, north Tajikistan, and southeastern Kazakhstan) and South Central Asia (Afghanistan and Pakistan); and subpopulation 7 includes accessions from northwestern Jordan and around the Mediterranean Sea basin in Israel/Palestinian Authority Territories, Cyprus, and northern Libya.

### Phenotypic analyses

The controls reacted as expected based on many previous experiments (Steffenson *et al.* 2017). Hiproly, the susceptible control, exhibited high ITs ranging from 3 to 3+ to all *P*. *graminis* cultures. Chevron (with *Rpg1*) exhibited predominantly low ITs to pathotypes MCCFC (0; to 1) and HKHJC (1 to 0;) and mostly moderate to high ITs (2 to 3−) to TTKSK, QCCJB, and 92-MN-90. Accessions carrying the gene complex of *rpg4*/*Rpg5* (Q21861 and Q/SM20) exhibited low ITs (0;1 to 1,2) to cultures TTKSK, QCCJB, MCCFC, and 92-MN-90 and moderately high ITs (3−) to HKHJC. Uniform infection levels were observed on the WBDC accessions across all experiments, facilitating the unambiguous scoring of the raw ITs (Table S1).

Genetic variance, error variance, and broad-sense heritability estimates were calculated for the reactions of the WBDC to all stem rust cultures (Table 1). Heritabilities were moderate to high, ranging from 0.41 for *Pgt* pathotype HKHJC to 0.82 for *Pgt* pathotype TTKSK. Numeric phenotype values (as given by the CI) of the WBDC to all *P. graminis* cultures were positively correlated with one another and very highly significant (*P* = 0.00001) (Table 2). The highest *r^2^* value (0.73) was found between *Pgt* pathotypes MCCFC and QCCJB and the lowest (0.25) between *Pgt* pathotype HKHJC and *Pgs* culture 92-MN-90.

**Table 1:** Estimated genetic variance (*σ_g_^2^*), error variance (*σ_e_^2^*), and broad-sense heritability (*H^2^*) for resistance to *Puccinia gramini*s f. sp. *tritici* pathotypes TTKSK, QCCJB, MCCFC, HKHJC and *Puccinia graminis* f. sp. *secalis* culture 92-MN-90 in the WBDC

*P. graminis* Culture	*σ_g_^2^*	*σ_e_^2^*	*H^2^*
TTKSK	0.19	0.21	0.82
QCCJB	0.37	0.40	0.74
MCCFC	0.18	0.15	0.79
HKHJC	0.05	0.15	0.41
92-MN-90	0.55	0.88	0.65

**Table 2:** Pearson correlation matrix for the mean coefficient of infection values of the WBDC to *P. gramini*s f. sp. *tritici* pathotypes TTKSK, QCCJB, MCCFC, HKHJC and *P. graminis* f. sp. *secalis* culture 92-MN-90

	TTKSK	QCCJB	MCCFC	HKHJC	92-MN-90
TTKSK	1	–	–	–	–
QCCJB	0.51	1	–	–	–
MCCFC	0.52	0.73	1	–	–
HKHJC	0.27	0.39	0.33	1	–
92-MN-90	0.48	0.64	0.67	0.25	1

All correlations are significant at *P* = 0.00001.

**Figure 5:**
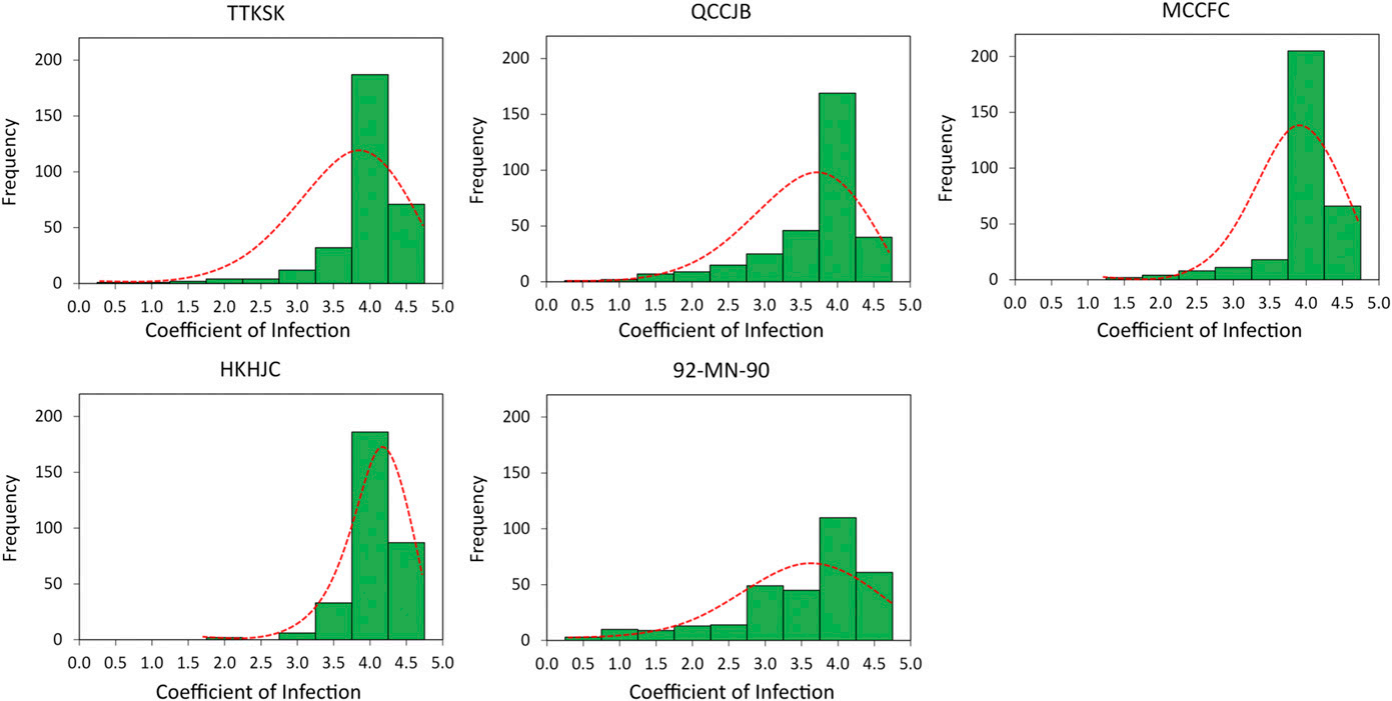
Phenotypic distribution of the coefficient of infection to *P. graminis* f. sp. *tritici* pathotypes TTKSK, QCCJB, MCCFC, and HKHJC and *P. graminis* f. sp. *secalis* culture 92-MN-90 in the WBDC.

A wide range of variation was found among the WBDC accessions in response to all five *P*. *graminis* cultures with the CI skewed mostly toward higher values, indicative of greater susceptibility (Figure 5). Numeric CI values ranged from 0.5 to 4.5 with mean of 3.8 to pathotype TTKSK; from 0.5 to 4.3 with mean of 3.5 to pathotype QCCJB; from 1.0 to 4.3 with mean of 3.8 to pathotype MCCFC; from 1.8 to 4.5 with mean of 3.9 to pathotype HKHJC, and from 0.5 to 4.4 with mean of 3.3 to isolate 92-MN-90. The frequency of resistant accessions (those with mean CI of 2.7 or less) in the WBDC was highest in response to *Pgs* isolate 92-MN-90 (19.4%), followed by *Pgt* pathotypes QCCJB (13.1%), TTKSK (5.4%), MCCFC (5.1%), and HKHJC (0.6%). Based on the resistance threshold of CI ≤ 2.7, there were 43 WBDC accessions resistant to one *P. graminis* culture, 10 resistant to two cultures, 14 resistant to three cultures, eight resistant to four cultures, and none resistant to all five cultures. The accessions with the widest resistance spectrum (*i.e.*, to three and four cultures) originated mainly from subpopulations 2, 6, and 7 (Table S1). Significant differences for the mean numeric CI were observed among the identified subpopulations to all *P. graminis* cultures (Table 3). Most notably, accessions comprising subpopulation 6 (primarily from Central Asia) exhibited the lowest numeric CI (*i.e.*, the most resistant reactions) to all stem rust cultures with the exception of HKHJC where subpopulation 2 had the lowest value (Figure 3B and Table 3).

**Table 3:** Number of accessions, mean, minimum, and maximum of seedling coefficient of infection means observed in each subpopulation (SP) of the WBDC to *P. gramini*s f. sp. *tritici* pathotypes TTKSK, QCCJB, MCCFC, HKHJC and *P. graminis* f. sp. *secalis* culture 92-MN-90

		TTKSK			QCCJB			MCCFC			HKHJC			92-MN-90		
SP	Number	Mean*^a^*	Minimum	Maximum	Mean	Minimum	Maximum	Mean	Minimum	Maximum	Mean	Minimum	Maximum	Mean	Minimum	Maximum
SP1	29	3.64ab	0.50	4.25	3.78a	2.83	4.17	3.96a	3.50	4.19	4.02ab	3.38	4.25	3.53ab	1.58	4.17
SP2	44	3.71ab	2.53	4.25	3.40ab	0.50	4.33	3.67ab	1.09	4.25	3.83b	1.83	4.50	3.05bc	0.98	4.25
SP3	17	3.96a	2.00	4.50	3.40ab	2.04	4.00	3.82ab	3.10	4.06	4.04ab	3.42	4.50	3.40abc	1.80	4.04
SP4	39	3.90a	2.55	4.50	3.71a	0.99	4.33	3.93a	2.95	4.25	3.98ab	2.79	4.50	3.73a	1.98	4.21
SP5	47	3.79a	1.81	4.50	3.74a	2.13	4.33	3.92a	3.13	4.25	3.92ab	2.75	4.50	3.61a	2.57	4.33
SP6	46	3.41b	1.64	4.50	3.14b	1.18	4.33	3.51b	1.51	4.06	3.88b	2.83	4.25	2.75c	0.50	4.17
SP7	57	3.77ab	1.00	4.50	3.45ab	1.25	4.33	3.77ab	1.50	4.25	3.91ab	2.75	4.50	3.22abc	0.63	4.38
SPN	35	3.85a	1.75	4.50	3.62a	1.30	4.33	3.91a	2.18	4.13	4.14a	3.75	4.50	3.24abc	0.84	4.21

a Means followed by different letters in the same column are significantly different using Tukey’s Honestly Significant Differences (HSD) (*α* = *0.05*).

### Molecular assays for the Rpg5 and Rpg1 stem rust resistance genes

In a recently published study, Steffenson *et al.* (2017) evaluated the seedling reaction of 2859 *Hordeum* accessions to pathotype TTKSK, including 301 accessions of the WBDC. Eleven WBDC accessions (WBDC014, WBDC032, WBDC119, WBDC209, WBDC213, WBDC214, WBDC220, WBDC224, WBDC225, WBDC302, and WBDC333) exhibited consistently resistant reactions across all experiments and were therefore considered likely candidates to carry the *rpg4*/*Rpg5* complex (Table S1). These 11 accessions plus four additional ones (WBDC116, WBDC125, WBDC345, and WBDC349) were considered likely candidates carrying the *rpg4*/*Rpg5* complex. To confirm whether these accessions might carry the *rpg4*/*Rpg5* complex, the molecular assay for a functional *Rpg5* gene was conducted on these 15 accessions. Fourteen of the 15 resistant WBDC accessions tested positive for a functional *Rpg5* gene (Mamo *et al.* 2015; Steffenson *et al.* 2017). The resistant accessions WBDC040 and WBDC174 were not tested for a functional *Rpg5* gene. Only two accessions (WBDC094 from Karak and WBDC238 from Amman in Jordan) exhibited low ITs to pathotype HKHJC in this study, a reaction suggestive of *Rpg1*-mediated resistance. However, molecular analysis revealed that both accessions were negative for a functional *Rpg1* gene (Steffenson *et al.* 2017).

### GWAS for P. graminis resistance in the WBDC and gene annotation

**Figure 6:**
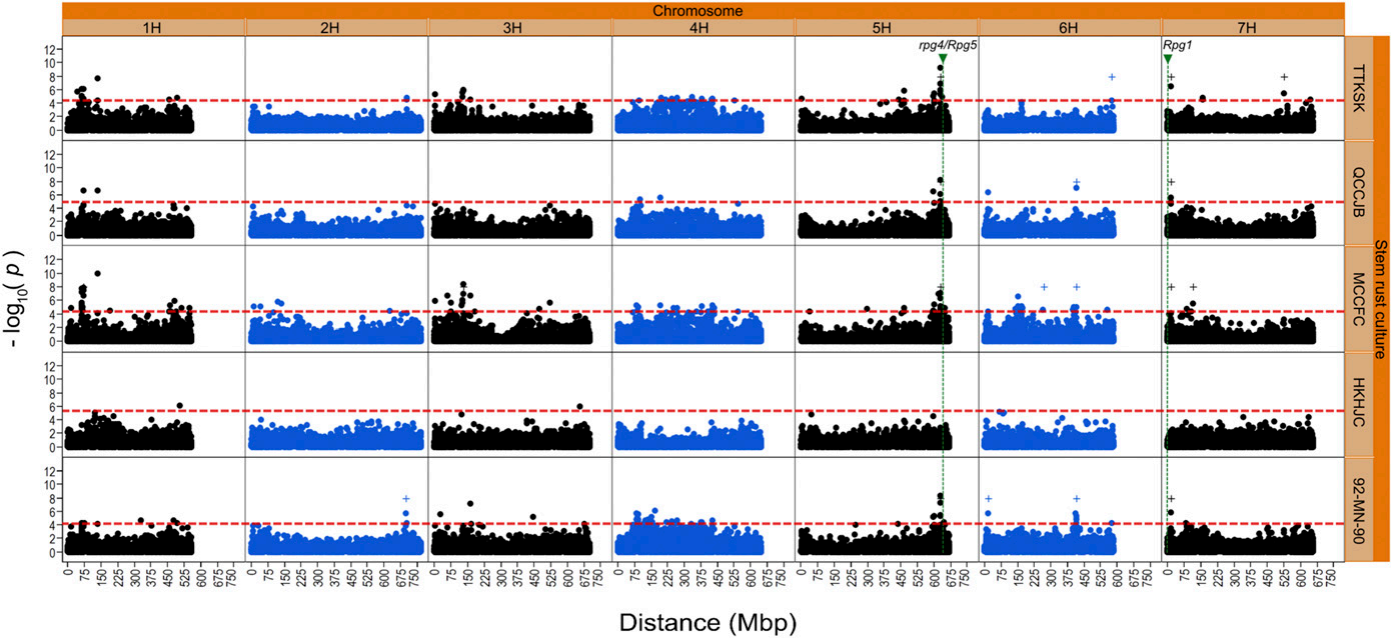
Manhattan plot displaying SNP markers significantly associated with resistance to four *P. graminis* f. sp. *tritici* pathotypes (TTKSK, QCCJB, MCCFC, and HKHJC) and one *P. graminis* f. sp. *secalis* culture 92-MN-90 in the WBDC. Two models were used: the *K* model (designated by circles) and the *G* model [designated by plus (+) signs]. All plotted *G* model hits are significant. The threshold for QTL detection for each *P. graminis* culture using the *K* model is shown with a horizontal dashed red line. Physical positions of the stem rust resistance genes *Rpg1* and *rpg4/Rpg5* are given on the map.

Statistical transformation of the CI values did not improve the normality of the data based on the Shapiro–Wilk test nor the identification of QTL; therefore, the GWAS results are based on the untransformed CI means. Marker–trait associations found for the *K* model were very similar to those found for the *Q* + *K* model (data not shown); thus, only the GWAS results for the *K* and *G* models are given. A total of 250 significant marker hits were identified in the WBDC for resistance to the five *P. graminis* cultures using the *K* model (Table S2). Using the physical distances and LD of adjacent significant markers within a given window, we identified the 45 most significant and unique QTL to one or more of the five *P. graminis* cultures using the *K* model (Figure 6 and Table 4). Nineteen of the 45 (42.2%) QTL identified using the *K* model were validated using the *G* model, providing support for the robustness of the associations. Twenty-nine QTL were associated with resistance to a single *P. graminis* culture, eight were associated with resistance to two cultures, five were associated with resistance to three cultures, and three were associated with resistance to four cultures. QTL associated with resistance to three or more *P*. *graminis* cultures are particularly noteworthy. Markers *S5H_596737839* (on chromosome 5H), *S5H_630137153* (chromosome 5H), and *S7H_14599947* (chromosome 7H) were significantly associated with the phenotypic response to four (TTKSK, QCCJB, MCCFC, and 92-MN-90) of the five *P*. *graminis* cultures. The associations of *S5H_596737839* with resistance to the four *P*. *graminis* cultures were detected using the *K* model only, whereas the associations with *S5H_630137153* and *S7H_14599947* were identified using both the *K* and the *G* models (Table 4). The amount of phenotypic variation explained by these three markers ranged from 10 to 15, 17 to 23, and 14 to 20%, respectively (Table 4). Markers *S1H_135744206* on chromosome 1H, *S4H_189407280* and *S4H_426125219* on 4H, and *S6H_14901366* and *S6H_415545279* on 6H were associated with resistance to three of the five *P*. *graminis* cultures (in no case with HKHJC) based on the *K* or *K* and *G* models. The phenotypic variation explained by QTL at these markers ranged from 15 to 25, 15 to 19, 11 to 18, 12 to 16, and 14 to 18%, respectively.

**Table 4:** Markers significantly associated with seedling resistance in the WBDC to *P. graminis* f. sp. *tritici* pathotypes TTKSK, QCCJB, HKHJC, MCCFC, and *P. graminis* f. sp. *secalis* culture 92-MN-90

Marker	MAF*^a^*	Alleles	TTKSK	QCCJB	MCCFC	HKHJC	92-MN-90	*R^2^**^b^*	POPSEQ Distance (cM)*^c^*	LD*^d^*	Gene Name*^e^*	Gene Function*^f^*	Resistance Source*^g^*
S1H_15633898	0.06	G/A	–	–	*+^K^**^i^*	–	–	0.09	27.06	**0.24**			SP2–SP6
S1H_71499376	0.05	G/A	–*	+^K^*	+^KG^*	–*	–*	0.14–0.24	46.4	**0.20**			SP6
S1H_74214978	0.07	A/G	–	–	–	–	*+^K^*	0.11	46.47	**0.29**			SP6
S1H_135744206	0.05	C/T	+^K^*	+^K^*	+^K^*	–*	–*	0.15–0.25	47.8	0.00			SP6
S1H_189554644*^h^*	0.05	T/C	–	–	*+^K^*	–	–	0.08	47.93	0.00			SP2
S1H_329009785	0.05	C/G	–	–	–	–	*+^K^*	0.17	48.19	0.01			SP6
S1H_482689791	0.05	T/A	–	–	*+^K^*	–	–	0.12	71.05	0.06			SP6
S1H_493058484	0.14	G/T	+^K^*	–*	–*	–*	+^K^*	0.09–0.11	80.27	0.01			SP6
S1H_503256550	0.10	G/A	–	–	–	*+^K^*	–	0.12	86.28				SP2
S2H_6224512	0.08	C/T	–	–	*+^K^*	–	–	0.06	5.31	0.12			SP2
S2H_118241413	0.07	T/C	–	–	*+^K^*	–	–	0.09	54.94	0.06			SP2
S2H_624272241	0.05	T/C	–	–	*+^K^*	–	–	0.04	67.68	0.00			SP1
S2H_698149422	0.14	C/T	–	–	–	–	*+^KG^*	0.14	105.54	0.00			SP6
S2H_704316447	0.14	C/T	*+^K^*	–	–	–	–	0.07	107.38		HORVU2Hr1G104640	U-box domain-containing protein	SP1
S3H_2871173	0.07	C/T	+^K^*	–*	+^K^*	–*	–*	0.17–0.22	2.03	0.05			SP6
S3H_28096251	0.12	G/T	–	–	–	–	*+^K^*	0.09	33.91	0.03			SP2–SP6
S3H_59710586	0.08	T/C	–	–	*+^K^*	–	–	0.18	45.86	**0.31**			SP2–SP6
S3H_130142435	0.05	A/T	+^K^*	–*	+^KG^*	–*	–*	0.16–0.27	48.72	**0.28**			SP2–SP3
S3H_161744326	0.11	G/C	–	–	–	–	*+^K^*	0.16	49.7	0.02			SP2–SP4–SP6
S3H_443833059	0.08	G/A	–	–	–	–	*+^K^*	0.07	51.91	0.10			SP2
S3H_521836324	0.06	C/T	–	–	*+^K^*	–	–	0.13	59.45	0.02			SP2
S3H_655238400	0.05	G/T	–	–	–	*+^K^*	–	0.05	127.31				SP2
S4H_84342294	0.05	G/A	–*	–*	+^K^*	–*	+^K^*	0.16–0.19	51.04	**0.60**			SP6
S4H_96878120	0.05	C/T	*+^K^*	–	–	–	–	0.14	51.32	**0.53**			SP6
S4H_100361862	0.08	T/G	–	*+^K^*	–	–	–	0.15	51.32	**0.37**			SP2–SP6
S4H_152479331	0.06	T/C	–	–	–	–	*+^K^*	0.17	51.38	**0.46**			SP6
S4H_166776967	0.06	G/C	–	–	–	–	*+^K^*	0.15	51.47	**0.70**			SP6
S4H_189407280	0.08	C/T	+^K^*	+^K^*	+^K^*	–*	–*	0.15–0.19	51.42	**0.80**			SP2–SP6
S4H_426125219	0.05	G/A	+^K^*	–*	+^K^*	–*	+^K^*	0.11–0.18	51.48				SP2–SP6
S5H_4727167	0.05	C/T	*+^K^*	–	–	–	–	0.13	7.42	0.07			SP2–SP6
S5H_299790192	0.05	G/C	–	–	*+^K^*	–	–	0.10	44.22	0.01			SP6
S5H_464225644	0.05	C/T	+^K^*	–*	+^K^*	–*	–*	0.06	50.01	0.02			SP6
S5H_596737839	0.06	C/T	+^K^*	+^K^*	+^K^*	–*	+^K^*	0.10–0.15	123.89	0.06	HORVU5Hr1G094700–HORVU5Hr1G094710	Disease resistance-responsive (dirigent-like protein) family protein	SP2–SP6–SP7
S5H_630137153	0.07	G/A	+^KG^*	+^KG^*	+^KG^*	–*	+^KG^*	0.17–0.23	143.5				SP6
S6H_14901366	0.07	A/T	–*	+^K^*	+^K^*	–*	+^KG^*	0.12–0.16	15.62	0.00			SP3–SP6
S6H_150096979	0.07	C/T	–	–	*+^K^*	–	–	0.06	53.58	0.15			SP3
S6H_267991768*^h^*	0.05	G/A	–	–	*+^KG^*	–	–	0.06	55.06	0.00			SP3
S6H_415545279	0.06	C/T	–*	+^KG^*	+^KG^*	–*	+^KG^*	0.14–0.18	60.02	0.05			SP6
S6H_553884761	0.06	G/C	–	–	*+^K^*	–	–	0.08	93.89	0.03			SP3
S6H_575766386*^h^*	0.06	G/A	+^KG^*	–*	–*	–*	+^K^*	0.11–0.12	117.87		HORVU6Hr1G091540	Protein kinase superfamily protein	SP6
S7H_14599947	0.05	C/T	+^KG^*	+^KG^*	+^KG^*	–*	+^KG^*	0.14–0.20	11.62	0.00			SP6
S7H_88235964	0.05	G/A	–	–	*+^K^*	–	*+^K^*	0.05–0.06	62.12	0.00			SP2
S7H_112969908	0.05	C/G	–	–	*+^KG^*	–	–	0.12–0.16	66.36	0.00	HORVU7Hr1G041160	Membrane attack complex component/perforin (MACPF) domain	SP6
S7H_156723880	0.46	G/A	*+^K^*	–	–	–	–	0.07	68.12	0.02	HORVU7Hr1G047080	MATE efflux family protein	SP6
S7H_526203164	0.05	G/C	*+^KG^*	–	–	–	–	0.12–0.16	77.4				SP6

a MAF, Minor allele frequency for SNP marker.

b R^2^, Proportion of phenotypic variance range explained by SNP marker.

c POPSEQ map distance in cM.

d Linkage disequilibrium (LD) estimated as *r^2^* between sequentially listed marker pairs within a chromosome (next marker in the list). Marker pairs having LD with *r^2^* ≥ 0.2 are in boldface.

e Gene name is according to barley genome annotation (http://webblast.ipk-gatersleben.de/barley_ibsc/downloads/).

f Gene function is listed for cases where the SNP marker is part of a gene implicated in disease resistance.

g Subpopulation source(s) for resistance alleles.

h Possible coincident QTL identified previously in the WBDC for QCCJC, MCCFC, and 92-MN-90 (Steffenson *et al.* 2007), where S1H_189554644 was coincident with QTL bpb-9717_58.7_MCCFC; S6H_267991768 was coincident with QTL bpb-4783_77.4_MCCFC; and S6H_575766386 was coincident with QTL bpb-2304/bpb-0403/bpb-7146_137_92-MN-90.

i +, SNP markers found to be significantly associated with *P. graminis* resistance using the *K* model (*K*), *G* model (*G*), or using both of the *K* and *G* models (*KG*). Significant association of multiple *P. graminis* cultures with the same markers is indicated by an asterisk (*).

With respect to individual stem rust cultures, the highest number of QTL identified was to MCCFC (27) and the lowest number was to HKHJC (2). The number of QTL identified for the other three *P*. *graminis* cultures were 16 for TTKSK, nine for QCCJB, and 18 for 92-MN-90. Considering individual barley chromosomes, nine QTL were identified on chromosome 1H, five on 2H, eight on 3H, seven on 4H, five on 5H, six on 6H, and finally five on 7H (Table 4). The amount of variation explained by any one QTL ranged from 4% (*S2H_624272241*) to 27% (*S3H_130142435*). Collectively, 30 QTL explained 10% or more of the phenotypic variation to any given *P. graminis* culture. Among the markers found significantly associated with resistance, the MAF ranged from 0.05 to 0.46 (Table 4). The highest frequency of resistance alleles to cultures TTKSK, QCCJB, MCCFD, and 92-MN-90 was from subpopulation 6, whose accessions mostly originated from Central Asia (Table 4). The highest frequency of resistance alleles in response to culture HKHJC was in subpopulation 2, whose accessions mostly originated from the Levant region (Table 4). LD estimates between significantly associated markers can be used to identify those pairs detecting the same QTL, thereby reducing redundant associations for resistance to a specific *P. graminis* culture. Among the 45 QTL identified, there were 11 cases of significant marker LD (*r^2^* ≥ 0.2) between sequentially listed marker pairs within a chromosome, which in most cases were associated with resistance to different *P. graminis* cultures (Table 4)

### Searching for candidate genes/QTL

Based on the recently published barley reference genome assembly, we identified five candidate genes associated with disease resistance. These five genes are HORVU2Hr1G104640, HORVU5Hr1G094700/HORVU5Hr1G094710, HORVU6Hr1G091540, HORVU7Hr1G041160, and HORVU7Hr1G047080 identified by the SNP markers *S2H_704316447*, *S5H_596737839*, *S6H_575766386*, *S7H_112969908*, and *S7H_156723880*, respectively. All these genes play a role in various defense responses, including the biosynthesis of lignin and cell death (Table 4).

## Discussion

### Resistance to P. graminis in the WBDC

Virulent cultures of *P. graminis* are a potential threat to barley production in many parts of the world, especially North America where most cultivars have been bred with the now-vulnerable *Rpg1* resistance. To counter these virulent populations of the pathogen, new sources of stem rust resistance are needed. Thus, the focus of this research was to evaluate a diverse collection of wild barley accessions for resistance to different *P. graminis* cultures, including those virulent for *Rpg1* at the seedling stage, and utilize GWAS to accurately position identified resistance loci based on the recently completed reference genome sequence of barley (Mascher *et al.* 2017). Resistance to TTKSK is of greatest concern to breeders presently given the pathotype’s recent emergence and wide virulence on barley germplasm. In several large screening studies of cultivated barley germplasm (*i.e.*, cultivars, breeding lines, and landraces), the frequency of resistance to pathotype TTKSK was found to be very low (Case 2017; Mamo *et al.* 2015; Steffenson *et al.* 2017; Zhou *et al.* 2014). The highest frequency of resistance found in any cultivated barley germplasm was with Swiss landraces. Steffenson *et al.* (2016) screened 73 barley landraces from eastern Switzerland for resistance to pathotype TTKSK and found 32 (43.8%) were resistant. Molecular analyses revealed that most (29 of 32) of these resistant landraces carried a functional *Rpg5* resistance gene. To widen the search for resistance to pathotype TTKSK, Steffenson *et al.* (2017) recently evaluated a highly diverse assemblage of 935 *H*. *vulgare* subsp. *spontaneum* accessions (301 from the WBDC and 634 from other collections) and identified only 13 (1.4%) with resistance. As was the case for cultivated germplasm, molecular analyses revealed that nearly all (11 of 13) of the resistant wild barley accessions carried a functional *Rpg5* resistance gene. Collectively, these studies demonstrate the paucity of resistance sources and resistance alleles in the primary genepool of barley to this widely virulent pathotype. In the current study, 17 (5.4%) of the 314 WBDC accessions exhibited resistance to TTKSK based on the mean CI (Table S1). In addition to pathotype TTKSK, the frequency of resistance to other *P*. *graminis* cultures was low to very low in the WBDC. Only 41 (13.1%), 16 (5.1%), 2 (0.6%), and 61 (19.4%) accessions were resistant to cultures QCCJB, MCCFC, HKHJC, and 92-MN-90, respectively. The extremely low frequency of resistance to pathotype HKHJC was notable; the two accessions found resistant were both from Jordan.

Accessions with resistance to multiple *P*. *graminis* cultures are of greatest interest in breeding. In this study, nine accessions exhibited resistance to two *P*. *graminis* cultures, 15 to three cultures and eight to four cultures (Table S1). Unfortunately, none of the WBDC accessions had resistance to all five cultures. Many of the accessions (15 out of 32) with the broadest resistance spectrum (to two or more rust cultures) were from subpopulation 6, originating from the Central Asian countries of Tajikistan, Turkmenistan, Uzbekistan, and Kazakhstan (Table 3). In some parts of Central Asia, stem rust epidemics occur with some frequency. Moreover, barberry (*Berberis* species), the alternate host of *P*. *graminis*, can be found infected in the region (Dawson *et al.* 2015; Steffenson *et al.* 2017), providing both a local source of inoculum and new virulence types via sexual recombination. The coevolution of wild barley with a persistent and diverse *P*. *graminis* population may have contributed to the high frequency of stem rust resistance in accessions from Central Asia. The Focused Identification of Germplasm Strategy (FIGS), developed by Mackay and Street (2004), aims to efficiently identify accessions with a target trait within a much reduced germplasm panel based on the analysis of various ecogeographic factors of collection sites. Given the results found in this investigation, additional collections of wild barley should be made in Central Asia to identify new stem rust resistance alleles.

### Population structure in the WBDC

A major limitation of GWAS is population stratification/structure. Differences in allele frequencies between subpopulations can result in falsely declared QTL. Several methods have been implemented to overcome this limitation including structured association mapping, unified mixed models, and step-wise GWAS that account for background marker effects of chromosomes not being tested for marker–trait associations (Pritchard *et al.* 2000; Yu *et al.* 2006; Bernardo 2013). Structure analysis of the WBDC was previously studied with a range of 6–10 subpopulations found (Steffenson *et al.* 2007; Fang *et al.* 2014; Ames *et al.* 2015). In the current study, population structure was performed using almost twice as many markers (4000 *vs.* 2359) as the most recent investigation on the WBDC (Ames *et al.* 2015). Moreover, since SNP markers generated by GBS were utilized, we eliminated the influence of ascertainment bias in the characterization of population structure in the WBDC and in the assignment of structure and genetic relatedness as cofactors in GWAS. Structure analysis resulted in the identification of seven subpopulations that varied in their allele frequencies (Figure 2). PCA confirmed this result by partitioning the WBDC accessions into the same seven differentiated subpopulations. Additionally, analysis of the genetic relationships among the WBDC accessions using genetic kinship identified the same population stratification (Figure 3). Not surprisingly, population structure was associated with the geographic distribution of the WBDC accessions. Accessions from Central Asia (subpopulation 6) were widely separated from those of west Asia (subpopulation 4) geographically, and both of these accession groups were separated from those of the Mediterranean Basin (subpopulation 7) as well as those of the Levant and southern Turkey (subpopulations 1, 2, 3, and 5). These results agree with a previous report documenting a relationship between population structure and geography in *H*. *vulgare* subsp. *spontaneum* (Russell *et al.* 2016).

### Linkage disequilibrium

The LD estimates found in the current study (0.10) were higher than those found in a previous study of the WBDC using markers generated by the Diversity Arrays Technology (DArT-Seq) method (0.02) (Ames *et al.* 2015). Previous reports demonstrated that LD extends over a long range in cultivated barley, a narrower range in landraces, and a very short range in wild barley (Morrell *et al.* 2005; Caldwell *et al.* 2006; Sallam *et al.* 2015). LD analysis of the WBDC in this study revealed low levels of association between neighboring markers; however, such low levels of LD are still sufficient to detect significant QTL, even for complex traits (Ersoz *et al.* 2009). Wild barley has a much lower level of LD than its cultivated forms due to the accumulation of outcrossing events throughout the long evolution of the species, the relatively recent shift from an outcrossing to a selfing mating system, and the higher frequency of recombination breakpoints (Morrell *et al.* 2005). The low levels of LD present in wild barley can be exploited for high-resolution mapping to identify and characterize candidate genes of interest (Caldwell *et al.* 2006).

### GWAS for P. graminis resistance in the WBDC

This study is the first to utilize the newly completed barley reference genome assembly for GWAS, and a large number (250) of significant markers were associated with resistance to five stem rust cultures based on 50,842 SNP markers using the *K* model. This large number of associations is unusual, but not unprecedented. For example, Gao *et al.* (2016) detected 333 significant markers in a panel of 381 spring wheats based on 90,000 SNP markers. Using the physical distances of adjacent significant markers and LD between markers within a given window, we identified the 45 most significant and unique associations (*i.e.*, QTL) (Figure 6 and Table 4). In several cases, two significant markers on the same chromosome were found in high but not perfect (*r^2^* < 1) LD with one another (Table 4). Interestingly, most of the markers in LD with each other had different resistance spectra. These results suggest either two closely linked genes or possibly alleles. Of the 45 identified QTL, 19 were validated using the *G* model (Figure 6 and Table 4). Detection of such a large number of QTL was likely due to the high heritability of the trait, the large number of markers identified across the genome through the GBS platform, and the appropriate modeling for population stratification and background marker effects. The power of QTL detection is a function of trait heritability (Lande and Thompson 1990; Bradbury *et al.* 2011). In this study, the heritabilities for seedling resistance were moderate to high (0.41–0.82) (Table 1) due to the careful standardization of inoculation techniques, rigorous control of environmental conditions during the experiments, and repeated phenotyping to achieve solid consensus data for infection types. The strength of LD is an important consideration in determining the resolution of association mapping. The WBDC was genotyped previously using several marker systems, including Diversity Arrays Technology (DArT) that yielded 1088 markers (Steffenson *et al.* 2007; Roy *et al.* 2010; Ames *et al.* 2015), oligonucleotide pool assays (BOPA1/BOPA2) that yielded 2374 SNP markers (Close *et al.* 2009; Roy *et al.* 2010; Ames *et al.* 2015), and DArT-Seq that yielded 7613 markers (Cruz *et al.* 2013; Ames *et al.* 2015). Using GBS, the number of markers generated (50,842) was >6 times that found for DArT-Seq (Ames *et al.* 2015). Using a sliding window of 25 markers, LD in the WBDC was 0.019, 0.024, and 0.022 based on 1088 DArT, 2374 BOPA1/BOPA2, and 7613 DArT-Seq markers, respectively, in comparison to 0.100 based on the 50,842 markers for the GBS platform. In this study, the increased number of markers generated for the WBDC facilitated the capture of more genetic variants, thereby enabling the detection of additional QTL (Steffenson *et al.* 2007; A. H. Sallam and B. J. Steffenson, unpublished data). Bradbury *et al.* (2011) assessed the power of QTL detection by GWAS using SNP data (BOPA1/BOPA2) and simulated phenotypic data in barley. They found that association models that included genetic kinship (*K* model) were superior to other models that included population stratification information such as PCA or subpopulation membership of structure analysis. For population structure-based association mapping, we found that the model accounting for genetic kinship among accessions (*i.e.*, the *K* model) was more efficient in identifying QTL than the model accounting only for population stratification using PCA (data not shown) and was similar in efficiency to the model accounting for both population stratification and genetic kinship (*i.e.*, *Q* + *K* model). Unlike genetic kinship, population stratification using STRUCTURE or PCA only considers a portion of the total genetic relatedness among accessions. This can result in an incomplete accountability of genetic relationships in GWAS panels (Kang *et al.* 2008).

### Resistance to P. graminis cultures QCCJB, MCCFC, and 92-MN-90 in the WBDC

Comparative analyses of previous stem rust resistance mapping studies are important for assessing whether possible new resistance loci have been detected or alternatively for providing some validation of previously identified resistance loci. A preliminary GWAS of the WBDC was conducted for resistance to *P. graminis* cultures QCCBJ, MCCFC, and 92-MN-90 using only 1088 DArT markers (Steffenson *et al.* 2007). The current investigation used the same raw IT data for these three *P*. *graminis* cultures as Steffenson *et al.* (2007), but with the added datasets to *Pgt* pathotypes TTKSK and HKHJC. Moreover, there were a number of other important advances made in the present analysis that were not included in the previous GWAS by Steffenson *et al.* (2007): (i) the raw IT data were converted into numeric phenotype values (CI) according to the methods of Zhou *et al.* (2014) to capture more genetic variation and be biologically more meaningful; (ii) using GBS, >46 times as many markers were used (1088 *vs.* 50,842), thereby providing much greater coverage across the entire genome; and (iii) using both the *K* and *G* models, a more comprehensive analysis of population stratification and genetic relatedness was achieved in the current study. In the investigation by Steffenson *et al.* (2007), 19 QTL that conferred resistance to one or more of *P. graminis* cultures QCCJB, MCCFC, and 92-MN-90 were identified. Of these 19 reported QTL, five were confirmed in the current investigation by lieu of their coincident mapping locations as best could be determined using the different marker datasets (Table 4). The different QTL found between this study and that reported by Steffenson *et al.* (2007) were likely due to advances in the analysis discussed above. Of the 19 resistance QTL identified by Steffenson *et al.* (2007), four were associated with QCCJB only, nine with MCCFC only, five with 92-MN-90 only, and one with pathotype MCCFC and also 92-MN-90.

A common limitation of GWAS is the inability of identified QTL to explain most of the phenotypic variability (Manolio *et al.* 2009). This is known as missing heritability and was suggested to be due to the failure of detecting rare variants (Manolio *et al.* 2009). Increasing the number of markers in a GWAS panel can increase LD, allowing for better detection of genetic variants (Manolio *et al.* 2009; Brachi *et al.* 2011). In this study, the >46-fold increase in marker numbers in the WBDC resulted in more markers being identified in LD with each other and with neighboring QTL. The increase of LD ultimately led to the capture of more genetic variants in this study than could be accounted for using the limited number of markers of the previous studies on the WBDC (Ames *et al.* 2015; Roy *et al.* 2010; Steffenson *et al.* 2007).

### Resistance to Pgt pathotypes TTKSK and HKHJC in the WBDC

Previous mapping studies identified QTL for seedling stage resistance to *Pgt* pathotype TTKSK in most barley chromosomes (Moscou *et al.* 2011; Mamo 2013; Mamo *et al.* 2015; Case 2017). In this study, two novel QTL were identified for resistance to pathotype TTKSK in chromosome 1H. One of these QTL was also associated with resistance to pathotypes QCCJB and MCCFC and tagged by the SNP marker *S1H_135744206*. The other QTL was mapped on the long arm of 1H and associated with resistance to both TTKSK and 92-MN-90, tagged by the SNP marker *S1H_493058484* (Table 4). In a transcriptome analysis study, Moscou *et al.* (2011) identified a QTL for TTKSK seedling resistance on the long arm of 1H in the Q21864/SM89010 double haploid mapping population. Additionally, a novel QTL that provides resistance to pathotype HKHJC was identified on the long arm of 1H linked to the SNP marker *S1H_503256550*. On chromosome 2H, a QTL was mapped at 193.5 cM that was associated with seedling resistance to TTKSK in the Q21861/SM89010 double haploid population (Moscou *et al.* 2011). This same chromosome was also found to harbor two seedling resistance QTL to pathotype TTKSK at 41.7 and 172.1 cM in a panel of Ethiopian/Eritrean barley landraces (Mamo 2013). In the current study, we identified a QTL for TTKSK seedling resistance associated with *S2H_704316447* on 2H at 107.4 cM. It is not known whether this QTL mapped at a coincident location to the previously reported QTL on 2H. Future investigations using sequence analysis of these markers will help to resolve this question. In chromosome 3H, two resistance QTL were identified in Swiss landraces, Ethiopian/Eritrean landraces, and wild barley accessions based on GWAS mapping and biparental studies (Mamo 2013; Mamo *et al.* 2015). In the current investigation, two QTL for TTKSK resistance were identified in chromosome 3H (*S3H_2871173* and *S3H_130142435*) that are not coincident with previously identified QTL, and both were also associated with MCCFC resistance. A novel QTL associated with resistance to HKHJC only was identified in 3H linked to SNP marker *S3H_655238400* (Figure 6 and Table 4). Additional genetic studies on the two novel QTL for HKHJC resistance are underway using biparental populations. A QTL for TTKSK resistance linked to BOPA marker *12_30995* was previously identified in chromosome 4H in Ethiopian/Eritrean barley landraces (Mamo 2013), whereas in the current study, two QTL were detected by the SNP markers *S4H_189407280* and *S4H_426125219*. These significant markers mapped 236.7 Mbp from each other despite the strong LD identified (0.80) and the close POPSEQ distance between them. This suggests that these markers may be detecting the same TTKSK resistance QTL. These TTKSK resistance QTL were also associated with resistance to two other *P. graminis* cultures. In this study, four QTL for resistance to TTKSK were identified in chromosome 5H, two of which were also associated with resistance to QCCJB, MCCFC, and 92-MN-90 and linked to SNP markers *S5H_596737839* and *S5H_630137153* (Figure 6 and Table 4). The SNP marker *S5H_596737839* was located in the long arm of 5H at 123.9 cM. At ∼33 Mbp (19.6 cM based on the POPSEQ distance) from this marker, *S5H_630137153* was identified as significantly associated with resistance to TTKSK, QCCJB, MCCFC, and 92-MN-90 using both the *K* and *G* models. Based on sequence analysis of the new barley reference genome assembly, *rpg4*/*Rpg5* is positioned at ∼640 Mbp in the long arm of 5H (150 cM based on POPSEQ map distance), ∼10 Mbp distal from *S5H_630137153* (Figure 6). Additionally, sequence comparison of *S5H_630137153* with *rpg4* and *Rpg5* revealed no significant similarity. These results confirm the novelty of the resistance QTL linked to *S5H_596737839* and *S5H_630137153*. Despite its large phenotypic effect and unique resistance spectrum, significant QTL for the *rpg4/Rpg5* complex were not detected in the WBDC in this study. This was likely due to the low frequency of the functional *Rpg5* resistance allele in the panel as reported by Steffenson *et al.* (2017). Four QTL for TTKSK resistance were previously reported in chromosome 5H (Moscou *et al.* 2011; Mamo 2013; Case 2017). Moscou *et al.* (2011) identified a QTL that was revealed to be *rpg4/Rpg5* in the long arm of 5H at 146.8 cM in the Q2186/SM89010 population. In the United States Department of Agriculture-Agricultural Research Service (USDA-ARS) Barley Core Collection, Case (2017) identified another QTL at 172.2 cM, linked to the SNP marker 11_10236. Moreover, on the short arm of 5H, we identified a QTL tagged by SNP marker *S5H_4727167* that is coincident with the one reported by Mamo (2013) and linked to *SCRI_RS_10929* based on mapping distance. In chromosome 6H, one QTL for TTKSK seedling resistance was mapped in the long arm and was linked to SNP marker *S6H_575766386* (Figure 6 and Table 4). This marker was also associated with resistance to *Pgs* isolate 92-MN-90. This is a novel QTL because none of the previous investigations identified QTL for TTKSK resistance in 6H. Close to the telomeric region in the short arm of 7H, marker *S7H_14599947* was found associated with resistance to TTKSK, QCCJB, MCCFC, and 92-MN-90 (Figure 6 and Table 4). This is also a novel resistance QTL and its detection with both GWAS models confirms the robustness of its effect. Additional studies should be made to validate the effect of these novel QTL and whether they confer useful resistance at the adult plant stage. No significant QTL signal was detected at the *Rpg1* locus in the short arm of 7H using GWAS. Although WBDC094 and WBDC238 from Jordan exhibited strong resistance to pathotype HKHJC (a hallmark reaction for the presence of *Rpg1*), the molecular assay indicated that the two accessions lacked a functional *Rpg1* gene (Steffenson *et al.* 2017). Blasting the *Rpg1* sequence against the new barley reference genome placed the resistance gene at 3.2 Mbp in the telomeric region of the short arm of 7H (Figure 6). Two additional QTL for TTKSK resistance were identified in 7H, tagged by SNP markers *S7H_156723880* and *S7H_526203164*, the latter one detected using both the *K* and *G* models. Moscou *et al.* (2011) identified a TTKSK QTL at 11.2 cM in the short arm of 7H.

### Candidate genes

To determine whether genes encoding proteins with known resistance motifs were identified in this GWAS investigation, a 2-Mbp region surrounding each significant SNP was investigated. Gene annotation identified the SNP marker *S2H_704316447*, linked with TTKSK resistance on chromosome 2H, as part of the HORVU2Hr1G104640 gene that is associated with a U-box domain-containing protein. This protein has been implicated in a mechanism that initiates defense responses against pathogens (González-Lamothe *et al.* 2006). In the short arm of chromosome 5H, *S5H_596737839* identified a QTL conferring resistance to four of the five *P. graminis* cultures tested in this study. This marker is part of the HORVU5Hr1G094700/HORVU5Hr1G094710 genes, which encode disease resistance-responsive dirigent-like proteins. Dirigent proteins are present in many plant species and are involved in the biosynthesis of lignin for cell wall construction and as a defense mechanism against pathogens (Afzal *et al.* 2009; Subramanyam *et al.* 2013). The effect of dirigent proteins in response to pathogen attack has been reported in wheat, soybean, and conifers (Afzal *et al.* 2009; Subramanyam *et al.* 2013). In chromosome 6H, marker *S6H_575766386* is part of the HORVU6Hr1G091540 gene, which encodes a protein kinase superfamily protein. This protein family was previously shown to be associated with disease resistance in wheat and *Arabidopsis* (Gurung *et al.* 2014; Hiruma *et al.* 2011). In chromosome 7H, marker *S7H_112969908* is part of the HORVU7Hr1G041160 gene that is associated with resistance to pathotype MCCFC based on the *K* and *G* models. This gene is associated with a membrane attack complex and perforin (MACPF) domain that provides plants with immunity against pathogens by triggering cell death to hinder pathogen growth (Morita-Yamamuro *et al.* 2005). The 7H SNP marker *S7H_156723880*, associated with TTKSK resistance, is part of the gene HORVU7Hr1G047080, which was implicated with a multi-drug and toxin extrusion protein (MATE) domain. This protein family is involved in many functions in plants, including disease resistance (Chen *et al.* 2015). In a study that used a homolog of MATE in *Arabidopsis*, inoculation with *Pseudomonas syringae* resulted in an increase of enhanced disease susceptibility 5 (EDS5) gene expression, which, in turn, induces the biosynthesis of salicylic acid, a compound involved in disease resistance signaling (Delaney *et al.* 1994; Nawrath *et al.* 2002). Collectively, these genes are candidates for further study on the resistance of barley to stem rust.

### Implications for breeding

The development and sustainability of resistant cultivars requires the continual identification of new sources of resistance and introgression of resistance alleles into elite genetic backgrounds. In this study, potentially novel seedling resistance sources were identified, but in low frequency. Although a wild species, *H*. *vulgare* subsp. *spontaneum* can readily hybridize with cultivated barley, thereby facilitating the easy transfer of stem rust resistance genes. The pyramiding of major-effect resistance genes is one strategy that has proven effective in providing stable stem rust resistance in wheat. With the emergence of “Ug99 lineage” and other similar groups of widely virulent *Pgt* pathotypes, it is important that new pyramids of effective genes be developed to avoid future losses due to stem rust. Barley is particularly vulnerable to stem rust due to the paucity of major-effect resistance genes, a result also confirmed in this study with the wild progenitor *H*. *vulgare* subsp. *spontaneum*. Currently, the most expedient short-term strategy for breeding barley for stem rust resistance is to pyramid *Rpg1* with *rpg4*/*Rpg5*. This combination will protect the crop from the predominant virulence types described in the *P*. *graminis* population. The new seedling resistance loci identified in wild barley from this study may also enhance both the level and spectrum of resistance in barley cultivars; however, they must be rigorously tested in the field for adult plant resistance since this is the critical developmental period when stem rust first infects the crop and causes the most damage. Obtaining adult plant stem rust reactions in the field is difficult with raw wild barley accessions due to the fact that they are largely unadapted to grow under the conditions of the northern Great Plains region and do not readily head due to photoperiod sensitivity in the equatorial TTKSK screening site in Kenya (Steffenson *et al.* 2017). Thus, to assess the wild barley resistance alleles under field conditions, we are first transferring them into adapted barley lines.

The incorporation of adult plant resistance is another sound strategy for protecting cereal crops from rust diseases in the long term. Case (2017) studied the genetics of stem rust resistance in Heitpas-5 and GAW-79, the sources of genes *Rpg2* and *Rpg3*, respectively, and detected QTL conferring moderate to high levels of adult plant resistance in the field. These and other identified loci for adult plant resistance can enhance the level and durability of stem rust resistance in barley. For barley breeding programs focusing on quantitative adult plant resistance, genomic selection, which relies on estimating genome-wide marker effects, could be an efficient breeding strategy. Genomic selection was found to be effective for improving stem rust resistance in wheat (Rutkoski *et al.* 2015) and could be readily implemented in barley breeding programs.

## 

## Supplementary Material

Supplemental material is available online at www.g3journal.org/lookup/suppl/doi:10.1534/g3.117.300222/-/DC1.
