# Supplementary material for: Genome-Wide Association Mapping of Stem Rust Resistance in Hordeum vulgare subsp. spontaneum
Source: G3 (Bethesda). 2017 Aug 30;7(10):3491–507. doi: 10.1534/g3.117.300222 (PMC5633397; doi:10.1534/g3.117.300222)
Supplement: Supplementary file 3 [file 3491FileS1.docx]

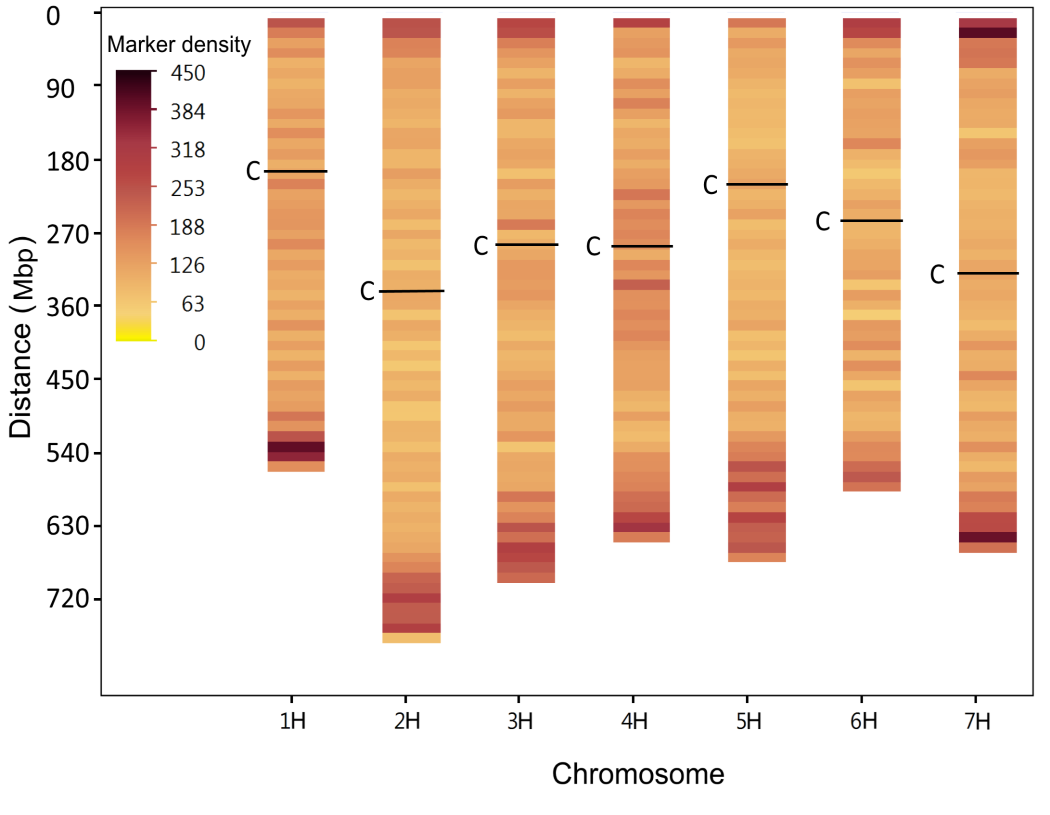


**Figure S1:** Distribution of the 50,842 SNP markers through the seven barley chromosomes with the barley assembly. SNP markers were ordered using the reference sequence “150831_barley_pseudomolecules.fasta” (Mascher *et al.* 2017). The heat cell key displays the density of the SNP markers on chromosomes.


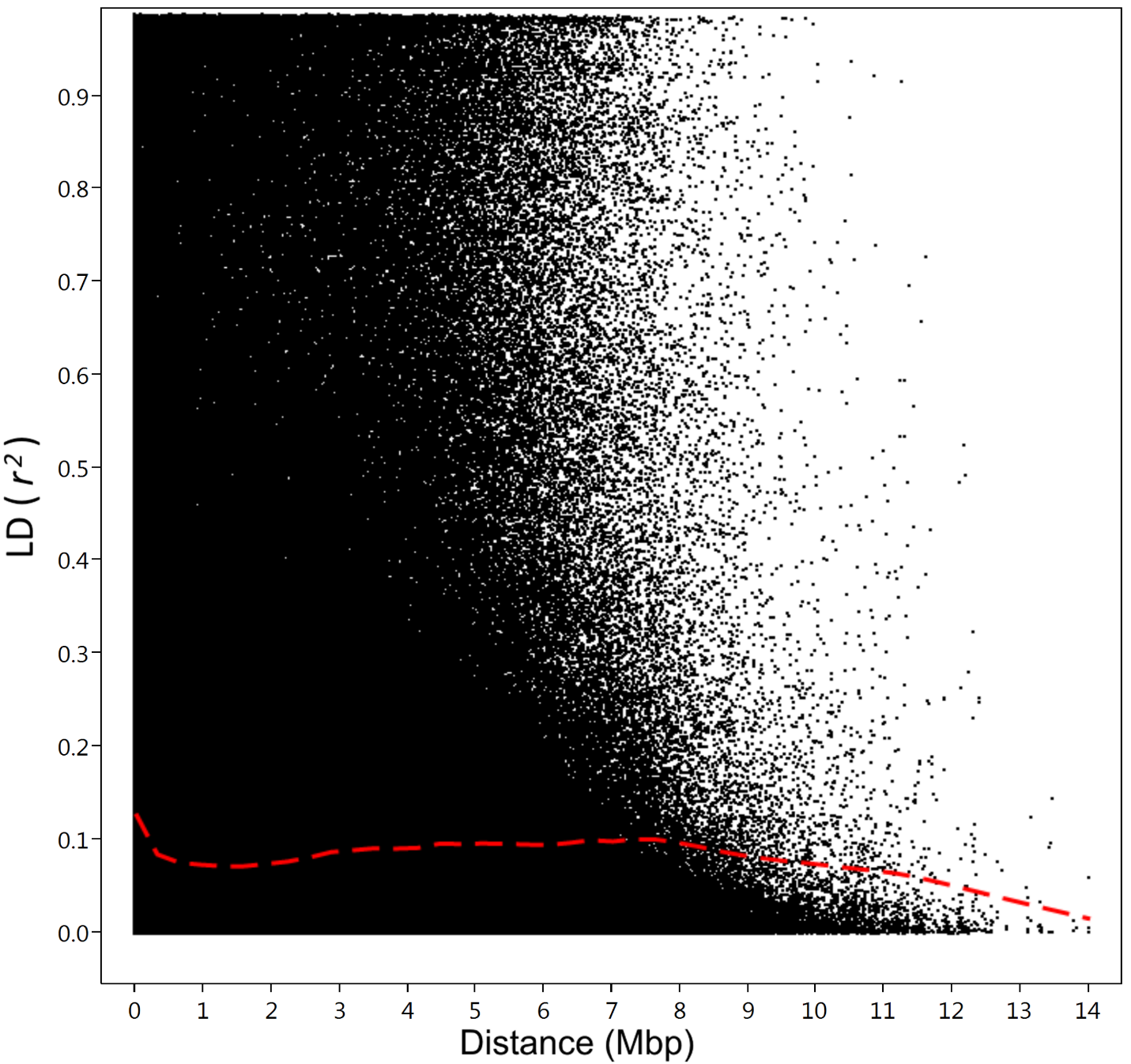


**Figure S2:** LD estimated as *r^2^* using a sliding window of 50 markers throughout the genome plotted against the physical distance. The dashed red curve represents LD decay presented as the local weighted scatter plot smoothing (LOWESS).
